# The relationships between social media use, time management, and decision-making styles

**DOI:** 10.3389/fpsyg.2025.1702767

**Published:** 2025-12-10

**Authors:** Hatice Şıngır

**Affiliations:** Division of Guidance and Psychological Counseling, Department of Educational Sciences, Gazi Faculty of Education, Gazi University, Ankara, Türkiye

**Keywords:** social media usage, time management, decision-making styles, undergraduate students, emerging adults

## Abstract

The present study examined the relationship between social media use, time management, and decision-making styles. The sample consisted of 612 participants, including 513 women (83.8%) and 99 men (16.2%), who were university students and young adults. Data were collected using a personal information form (age, gender, social media usage time, and academic achievement), the Time Management Scale, and the Melbourne Decision-Making Questionnaire. In addition to these measures, differences were analyzed concerning age, gender, academic achievement, and duration of social media use. Descriptive statistics, independent samples *t*-tests, one-way analysis of variance (ANOVA), chi-square tests, and regression analyses were employed in data analysis. The results indicated that female participants scored significantly higher than males on overall time management, time planning, and time wasters subscales. In contrast, male participants obtained significantly higher scores on the time attitude subscale. Furthermore, the self-esteem and hypervigilance subscales of the decision-making scale differed by gender; female participants reported lower self-esteem and higher hypervigilance scores compared to their male counterparts. Academic achievement was positively associated with time management skills, such that higher academic performance predicted better time management. Social media use was negatively and significantly associated with overall time management and all its subscales. In contrast, it was positively associated with buckpassing, procrastinatory, and hypervigilance decision-making styles and negatively associated with the careful decision-making style. A positive relationship was also identified between overall time management and decision-making styles. Specifically, individuals with better time management skills demonstrated higher self-esteem and a tendency toward careful decision-making, whereas negative associations were observed with buckpassing, procrastinatory, and hypervigilance styles. Finally, longer durations of social media use significantly predicted lower self-esteem, careful decision-making, and higher levels of buckpassing, procrastinatory, and hypervigilance decision-making styles.

## 1 Introduction

With the widespread adoption of digital technologies, habits related to information seeking, entertainment and communication have changed substantially. Recent international reports indicate that social media is used by a considerable majority of the world’s population and that many users spend approximately 2 h per day on these platforms (We Are Social and Meltwater, 2025). University students and young adults are among the groups that use social media most frequently. In particular, it has been suggested that the vast majority of Generation Z are in constant interaction with social media in their daily lives and that this situation may be associated with their decision-making behaviors (İnal, 2023). In other words, the decisions young people make, how they implement these decisions and how they structure their decision-making processes can be considered in relation to the time they spend on social media and their time management habits. Although social media facilitates access to information, spending intensive amounts of time in these environments may be related to how individuals use their time and how they manage decision-making processes.

Time management can be defined as individuals’ ability to plan and use their time efficiently in order to achieve their goals. It has been reported that effective time management is associated with academic achievement and is regarded as an important skill that supports academic performance (Britton and Tesser, 1991). The decision-making process, in which individuals evaluate alternatives and attempt to choose the most appropriate option (Byrnes, 2002), also involves self-regulatory skills related to time management, such as planning and the ability to direct attention toward a goal. Therefore, it is assumed that time management skills may co-occur with more planned and deliberate decision-making processes.

The efficient and planned use of time is evaluated not only as “controlling” time but also as a process associated with individuals’ quality of life (Alay and Koçak, 2003). Managing time is closely connected with individuals’ ability to regulate their own behaviors and activities (Karslı, 2004). At this point, the concept of self-regulation provides an explanatory framework for the theoretical grounding of the present study. Self-regulation is understood as individuals’ directing and monitoring their behaviors, thoughts and emotions in line with their goals (Bandura, 1991). From a behavioral perspective, self-regulation is related to how a person makes decisions about initiating or postponing an action, and about maintaining or terminating it (Eisenberg et al., 2010). It has been suggested that students with stronger self-regulation skills use their time more systematically and effectively and thus are better able to structure academic tasks and processes (Zimmerman, 2002). In a study conducted by Macan et al. (1990), students who perceived greater control over their time reported lower levels of stress. In contrast, attitudes such as indecisiveness, lack of planning, inability to say “no” and procrastination have been described as time traps that make time management more difficult for students (Kuşcu Karatepe et al., 2020).

Although patterns of time use may vary across cultures and societies (Gürbüz and Aydın, 2012), in societies that are considered more developed the importance of time has increasingly been emphasized, and with changing global conditions, the efficient and effective use of time has come to be regarded as necessary. The concepts of time and time management have been examined from different perspectives by many researchers (Alay and Koçak, 2003; Aslan et al., 2020; Britton and Tesser, 1991; Demirtaş and Özer, 2007; Erdem et al., 2005; Macan et al., 1990; Sarıkaya Aydın and Koçak, 2016; Yardım and Engin, 2022). In the present study, time management, decision-making styles and social media use are examined together in order to better understand the relationships among these variables.

Decision-making can be defined as the process by which individuals scan available options in order to select the most appropriate path to achieve a given goal. In other words, the decision-making process includes determining a goal, identifying options that may lead to that goal, evaluating and ranking these options, and choosing the one that appears most appropriate (Byrnes, 2002). However, individuals differ from one another in terms of their typical decision-making styles. For example, some individuals tend to make decisions carefully and confidently, whereas others may be more prone to postponing decisions, delegating responsibility to others, or making decisions in a highly aroused and hasty manner. Effective time management can be regarded as a functional skill in such decision-making processes that involve evaluating alternatives, analyzing risks and determining the most suitable option. Nevertheless, the number of studies that directly address the relationship between time management and decision-making styles appears to be limited. In a study examining the relationships between time management and decision-making processes, Varlamova (2008) found that time-management-related dimensions such as goal setting and perceived control over time were associated with more functional decision-making processes. This finding indicates a need for further studies that examine in more detail the relationships between time management and decision-making processes.

Social media use is another variable that is considered likely to be associated with individuals’ time management skills. Social media may contribute to information overload, frequent interruptions and distraction, thereby creating an environment in which it becomes more difficult to allocate time to structured tasks and to sustain attention on goal-directed activities. In studies on internet addiction and time management (Ali et al., 2024), it has been reported that as levels of internet/social media addiction increase, individuals tend to show weaker time management skills. In another study, Chukwu et al. (2022) reported that students generally had good time management skills, but that the direct effect of social media on time management was at a low level. These differing findings suggest that social media use may be related to time management, but that the nature and strength of this relationship may vary depending on cultural context, usage patterns and individual characteristics.

Considering time management skills together with decision-making processes and social media use is particularly important in terms of cognitive load and information flow in digital environments. In a study examining the effects of information overload on consumers’ decision-making processes on online platforms (Peng et al., 2021), intense information flow was found to be negatively associated with decision-making processes by straining individuals’ processing capacity. It has been suggested that individuals who encounter large amounts of information online may have difficulty making accurate and balanced decisions due to limited cognitive resources. From these perspectives, social media use, time management and decision-making styles can be conceptualized as interconnected aspects of self-regulation processes in contemporary digital environments. Findings related to social media, time management and decision-making processes indicate that these variables need to be examined together within the same study. The limited number of studies in which these three variables are investigated simultaneously increases the significance of the present study

This study examines the relationship between social media use, time management, and decision-making styles. Since studies addressing these variables in Turkey are limited, this research is expected to contribute to the literature. In line with this aim, the following research questions were posed:

- Do the scores of social media use, time management, and decision-making variables differ by age?
- Do the scores of social media use, time management, and decision-making variables differ by gender?
- Do the scores of social media use, time management, and decision-making variables differ by academic achievement?
- Do the scores of time management and decision-making variables differ according to social media use?
- Is there a relationship among social media use, time management, and decision-making variables?
- Do social media use, time management sub-dimensions, and decision-making style sub-dimensions predict one another?

## 2 Materials and methods

### 2.1 Research design

This study employed a correlational survey model to examine the relationships between social media use, time management, and decision-making styles. Correlational survey models are quantitative research designs that aim to determine the level of relationships among variables (Karasar, 2017).

### 2.2 Study group

The study was conducted with a total of 830 participants, consisting of university students and young adults from different faculties and departments in Türkiye, recruited through a self-selection sampling method. After excluding cases with missing, erroneous, or outlying data, the analyses were performed on 612 participants. Before carrying out the main analyses, the dataset was screened for missing values, inconsistent or implausible responses, and statistical outliers. Of the initial 830 cases, a total of 218 (26.3%) were excluded based on the predefined criteria described below. Data from 141 participants who provided incomplete demographic information or selected “other” or “prefer not to specify” for key demographic variables (e.g., age, educational level) were excluded because these responses did not allow us to classify them within the target population of university students and young adults. Information regarding the demographic characteristics of the participants is presented in Table 1.

**TABLE 1:** Demographic characteristics of participants.

Demographic information		n	%
Age	18–23	459	75.0
	24–36	153	25.0
	Total	612	100.0
Gender	Female	513	83.8
	Male	99	16.2
	Total	612	100.0
Education level	Undergraduate	594	97.1
	Postgraduate	18	2.9
	Total	612	100.0
Department	Quantitative	169	27.6
	Verbal	443	72.4
	Total	612	100.0
Class	1	89	14.5
	2	107	17.5
	3	67	10.9
	4	219	35.8
	Graduate	130	21.2
	Total	612	100.0
Academic success	2.00–3.00	217	35.5
	3.01–3.50	285	46.6
	3.51–4.00	110	18.0
	Total	612	100.0
Social media usage time	Time 2 or less	149	24.3
	2–4	249	40.7
	4–6	155	25.3
	More than 6 h	59	9.6
	Total	612	100.0

Table 1 shows that the participants were between 18 and 36 years old, and most of the study group reported using social media for 2–4 h per day. In addition, information regarding participants’ education levels, grade levels, academic departments, and academic achievement is also presented.

### 2.3 Data collection procedure

Ethical approval for this study was obtained from the Ethics Committee of Gazi University Rectorate (Approval No: 2025–438, Date: 17.03.2025). All participants provided informed consent online prior to participation. During the data collection process, participants were informed about the purpose of the study, and voluntary participation consent was obtained via an online consent form. All personal data were collected anonymously, and participant confidentiality was maintained throughout the research process. The scales used in the study were distributed to participants via social media.

First, a general screening of all data was conducted, and responses that were incomplete, erroneous, outliers, or deemed likely to compromise the overall consistency of the analyses were excluded from the study. Within this scope, data from 141 participants who selected “other” or “prefer not to specify” in the demographic information and data from 40 participants identified as outliers were removed. In addition, since age comparisons were planned between university students and young adults, excluding participants younger than 18 and older than 36 from the analyses was considered appropriate. Consequently, the analyses were carried out with data from 612 participants.

In the study, the Chi-Square Test was used for comparing two categorical variables, the Independent Samples *t*-test, a parametric method, was employed for comparing continuous variables with two categories that were normally distributed, and the One-Way ANOVA Test, a parametric method, was utilized for comparing continuous variables with more than two categories that were normally distributed. When statistically significant differences were found in comparisons of continuous variables with more than two categories, appropriate *post-hoc* tests were used to identify the differences between the variables. Pearson Correlation Analysis and Regression Analysis methods were applied to the responses given to the scales and their sub-dimensions.

A Normality Test was conducted to determine whether the responses given to the scales, their sub-dimensions, and duration of social media usage were normally distributed. When deciding whether the variables were normally distributed, skewness and kurtosis values were examined. According to Tabachnick and Fidell (2007), it is stated that the distribution of responses can be considered normal when skewness and kurtosis values are between -1.50 and +1.50.

The skewness values of the Time Management Scale (TMS) overall score and its sub-dimensions of time planning, time attitudes, and time wasters were found to be −0.099, −0.102, −0.007, and −0.413, respectively; while the kurtosis values were found to be −0.207, −0.394, 0.344, and 0.447, respectively. These values indicate that the TMS and its sub-dimensions are quite close to normal distribution.

The Melbourne Decision Making Scale (MDMQ) overall score has a skewness value of 0.321 and a kurtosis value of -0.104. Among the sub-dimensions of the MDMQ, the skewness values were calculated as -0.789 for the self-esteem sub-dimension, -1.135 for the vigilant decision-making sub-dimension, 0.577 for the Buckpassing sub-dimension, 0.344 for the procrastinative sub-dimension, and 0.175 for the hypervigilance sub-dimension. The kurtosis values for these sub-dimensions are 0.044, 0.801, -0.419, -0.786, and -0.835, respectively. Only the vigilant sub-dimension’s skewness (-1.135) and kurtosis (0.801) values approach the ± 1 limit. However, these values are still acceptable and indicate no serious violation of normality in the dataset.

The skewness and kurtosis values for the Social Media Usage (SMU) variable are -0.340 and -0.709, respectively. These values also indicate that the distribution can be considered normal.

In conclusion, the skewness and kurtosis values for all variables were found to be within the ± 1 range or relatively close to this limit; therefore, it can be said that the data largely meet the normal distribution assumption.

### 2.4 Data collection instruments

#### 2.4.1 Time Management Scale

The scale developed initially by Britton and Tesser (1991) to measure time management skills of university students was adapted into Turkish by Alay and Koçak (2002). While the original scale consisted of 35 items, the Turkish-adapted version comprised 27 items. It has three sub-dimensions: Time Planning (items 1, 2, 3, 4, 5, 6, 7, 8, 9, 10, 11, 12, 13, 14, 15, 16), Time Attitudes (items 17, 18, 19, 20, 21, 22, 23), and Time Wasters (items 24, 25, 26, 27). Higher total scores indicate positive attitudes toward time management. The scale is scored using a 5-point Likert-type scale, with responses ranging from always (5) to never (1). Examples of items in the scale include “Do you spend time planning each day?” and “Do you set a series of goals for an academic semester?” In the validity and reliability study for Turkey, the Cronbach’s Alpha coefficient was found to be 0.87 for the overall scale, 0.88 for the “Time Planning” dimension, 0.66 for the “Time Attitudes” dimension, and 0.47 for the “Time Wasters” dimension. For this study, the Cronbach’s Alpha coefficient was found to be 0.87 for the overall scale, 0.88 for the “Time Planning” dimension, 0.64 for the “Time Attitudes” dimension, and 0.44 for the “Time Wasters” dimension.

### 2.4.2 Melbourne Decision Making Scale

The scale (MDMQ I-II) developed by Mann et al. (1997) was adapted into Turkish by Deniz (2004). MDMQ I is a sub-dimension consisting of 6 items to determine self-esteem in decision-making. In this sub-scale, items 2, 4, and 6 are reverse-scored. MDMQ II consists of 22 items and measures (vigilant, buckpassing, procrastinative, and hypervigilance) decision-making styles. The scale consists of a total of 28 items as MDMQ I-II. Examples of items in the scale include “I postpone decision-making” and “I trust my decision-making ability.” In the scale, items 2, 4, 6, 8, 12, 16 constitute the vigilange sub-dimension; items 3, 9, 11, 14, 17, 19 constitute the buckpassing sub-dimension; items 5, 7, 10, 18, 21 constitute the procrastinatination sub-dimension; and items 1,13,15,20,22 constitute the hypervigilance sub-dimension. Responses on the scale are given as Not true (0), Sometimes true (1), and True (2).

The Cronbach’s alpha values of the sub-dimensions of the scale range between 0.65 and 0.80. For this study, the Cronbach’s Alpha coefficient was found to be 0.72 for the overall scale, 0.79 for the “Self-esteem” dimension, 0.79 for the vigilant dimension, 0.82 for the buckpassing dimension, 0.82 for the procrastinative dimension, and 0.79 for the hypervigilance dimension.

### 2.4.3 Social media usage

Participants indicated their social media usage duration in four categories (<2, 2–4, 4–6, >6 h).

## 3 Findings

### 3.1 Social media usage duration by age

In the study, the Chi-square test was used to examine whether there was a difference in social media usage duration in terms of age groups. As a result of the analysis, it was found that the duration of social media usage differed statistically significantly according to age groups. Chi-square test revealed a significant association between age groups and social media usage duration [χ^2^(3) = 34.984, *p* < 0.001, Cramer’s *V* = 0.239], indicating a small to medium effect size.

The distribution of age groups in terms of social media usage duration is presented in Table 2 as frequency and percentage.

**TABLE 2:** Social media usage duration by age (*N* = 612).

Age group	≤2 h	2–4 h	4–6 h	>6 h	Total
18–23	89 (19.4%)	187 (40.7%)	127 (27.7%)	56 (12.2%)	459 (100%)
24–36	60 (39.2%)	62 (40.5%)	28 (18.3%)	3 (2.0%)	153 (100%)
Total	149 (24.3%)	249 (40.7%)	155 (25.3%)	59 (9.6%)	612 (100%)

Values represent frequency and row percentages. Column percentages are reported in text.

Examining Table 2, 40.7% of participants in the 18–23 age group reported using social media for 2–4 h per day, 27.7% for 4–6 h, 19.4% for less than 2 h, and 12.2% for more than 6 h. In the 24–36 age group, 40.5% reported using social media for 2–4 h, 39.2% for less than 2 h, 18.3% for 4–6 h, and 2.0% for more than 6 h per day.

### 3.2 *T*-test Results of TMS and MDMQ sub-dimension scores by age

The *t*-test findings conducted to determine whether TMS and MDMQ sub-dimensions differ according to age are shown in Table 3.

**TABLE 3:** *T*-test results for differences in TMS and MDMQ sub-dimension scores by age.

Scale and sub-dimensions	Age	*n*	*M*	SD	*t*	*p*
TMS total	18–23	459	3.11	0.52	-3.65	< .001
	24–36	153	3.29	0.57		
Time planning	18–23	459	3.08	0.66	-3.08	.002
	24–36	153	3.30	0.77		
Time attitudes	18–23	459	3.12	0.61	-2.69	.007
	24–36	153	3.27	0.59		
Time wasters	18–23	459	3.19	0.67	-1.70	.090
	24–36	153	3.30	0.74		
MDMQ self-esteem	18–23	459	1.42	0.46	-1.55	.121
	24–36	153	1.48	0.46		
MDMQ vigilance	18–23	459	1.61	0.40	-2.13	.034
	24–36	153	1.69	0.37		
mDMQ Buckpassing	18–23	459	0.71	0.53	0.89	.375
	24–36	153	0.67	0.52		
mDMQ Procrastination	18–23	459	0.87	0.58	1.48	.139
	24–36	153	0.79	0.56		
MDMQ hypervigilance	18–23	459	0.94	0.47	2.22	.027
	24–36	153	0.83	0.58		

TMS, Time Management Scale; MDMQ, Decision-Making Style Scale; M, Mean; SD, Standard Deviation.

Examining Table 3, it can be seen that the total score of the Time Management Scale and all of its subdimensions, except for time wasters, differ significantly by age (*p* < 0.05). The results indicate that time management scores tend to increase as participants’ age increases. Participants in the 24–36 age group had significantly higher overall time management scores than those in the 18–23 age group (*t* = -3.650, *p* < 0.001, *d* = 0.34, small-to-medium effect size). Similarly, a significant difference was observed in the time planning subdimension (*t* = -3.306, *p* = 0.001, *d* = 0.31, small-to-medium effect size).

Only the vigilant and hypervigilant (panic) subdimensions of the MDMQ were found to differ significantly by age (*p* < 0.05). Participants in the 24–36 age group had significantly higher scores on the vigilant decision-making subdimension (*t* = -2.130, *p* = 0.034, *d* = 0.20). Conversely, participants in the 18–23 age group had significantly higher scores on the hypervigilant (panic) decision-making subdimension (*t* = 2.224, *p* = 0.027, *d* = 0.21).

### 3.3 Social media usage duration by gender

The chi-square test of independence conducted to examine the association between gender and social media usage duration was significant [χ^2^(3) = 8.81, *p* = 0.032, Cramer’s *V* = 0.12], indicating a small effect size.

The distribution of social media usage duration by gender in terms of frequency and percentage is presented in Table 4.

**TABLE 4:** Social media usage duration by gender.

Gender	Less than 2 h	2–4 h	4–6 h	More than 6 h	Total
Female	115 (22.4%) [77.2%]	209 (40.7%) [83.9%]	139 (27.1%) [89.7%]	50 (9.7%) [84.7%]	513 (100%)
Male	34 (34.3%) [22.8%]	40 (40.4%) [16.1%]	16 (16.2%) [10.3%]	9 (9.1%) [15.3%]	99 (100%)
Total	149 (24.3%) [100%]	249 (40.7%) [100%]	155 (25.3%) [100%]	59 (9.6%) [100%]	612 (100%)

Values are reported as *n* (column %) [row %].

Examining Table 4, it can be seen that men most frequently reported using social media for less than 2 h or for 2–4 h per day, whereas women most frequently reported using social media for 2–4 h or 4–6 h per day.

### 3.4 *T*-test results of TMS and MDMQ sub-dimension scores by gender

The *t*-test results conducted to determine whether TMS and MDMQ and sub-dimensions differ according to gender are presented in Table 5.

**TABLE 5:** *T*-test results for differences in TMS and MDMQ sub-dimension scores by gender.

Scale and sub-dimensions	Gender	*n*	*M*	SD	*t*	*P*
TMS total	Female	513	3.18	0.55	2.14	0.033
	Male	99	3.05	0.47		
Time planning	Female	513	3.17	0.70	2.71	0.007
	Male	99	2.97	0.66		
Time attitudes	Female	513	3.13	0.62	-2.21	0.028
	Male	99	3.28	0.55		
Time wasters	Female	513	3.27	0.68	3.80	<0.001
	Male	99	2.98	0.69		
MDMQ self-esteem	Female	513	1.40	0.47	-3.73	<0.001
	Male	99	1.59	0.39		
MDMQ vigilance	Female	513	1.63	0.40	-1.05	0.297
	Male	99	1.67	0.37		
MDMQ buckpassing	Female	513	0.72	0.53	1.57	0.116
	Male	99	0.63	0.51		
MDMQ procrastination	Female	513	0.87	0.59	1.31	0.191
	Male	99	0.78	0.51		
MDMQ hypervigilance	Female	513	0.95	0.55	3.89	< 0.001
	Male	99	0.72	0.53		

TMS, Time Management Scale; MDMQ, Decision-Making Style Scale; M, Mean; SD, Standard Deviation. Results are considered significant at *p* < 0.05.

As shown in Table 5, the total score of the Time Management Scale and all of its subdimensions differ significantly by gender (*p* < 0.05). Female participants reported significantly higher scores on overall time management [*t*(610) = 2.14, *p* = 0.033, *d* = 0.23, small effect], time planning [*t*(610) = 2.71, *p* = 0.007, *d* = 0.30, small effect], and time wasters [*t*(610) = 3.80, *p* < 0.001, *d* = 0.42, small-to-medium effect]. In contrast, male participants obtained significantly higher scores on the time attitudes subdimension [*t*(610) = 2.21, *p* = 0.028, *d* = 0.24, small effect].

It was observed that the self-esteem and Hypervigilance dimensions of the Decision-Making Styles Questionnaire (MDMQ) differed statistically significantly by gender (*p* < 0.001). Female participants had significantly lower self-esteem scores [*t*(610) = -3.73, *p* < 0.001, *d* = 0.41, small effect size] and significantly higher Hypervigilance scores [*t*(610) = 3.89, *p* < 0.001, *d* = 0.43, small effect size] compared to males.

### 3.5 Social media usage duration by academic achievement

The study examined whether social media usage duration differed by academic achievement using a Chi-square test. The analysis revealed that social media usage duration did not differ statistically significantly based on academic achievement [χ^2^(3) = 4.78, *p* = 0.572].

The distribution of social media usage duration by academic achievement is presented in frequency and percentage in Table 6.

**TABLE 6:** Social media usage duration by academic achievement (row % and column %).

Academic achievement	Less than 2 h	2–4 h	4–6 h	More than 6 h	Total
2.00–3.00	53 (35.6%, 24.4%)	80 (32.1%, 32.1%)	59 (38.1%, 38.1%)	25 (42.4%, 42.4%)	217 (35.5%)
3.01–3.50	67 (45.0%, 30.0%)	125 (50.2%, 50.2%)	66 (42.6%, 42.6%)	27 (45.8%, 45.8%)	285 (46.6%)
3.51–4.00	29 (19.5%, 13.0%)	44 (17.7%, 17.7%)	30 (19.4%, 19.4%)	7 (11.9%, 11.9%)	110 (18.0%)
Total	149 (100%)	249 (100%)	155 (100%)	59 (100%)	612 (100%)

No significant difference was observed in social media usage duration across academic achievement levels.

### 3.6 One-WAY ANOVA results of TMS and MDMQ sub-dimension scores by academic achievement level

The findings of the One-Way ANOVA test conducted to examine whether the scores of the Time Management Scale (TMS), the Decision-Making Styles Questionnaire (MDMQ), and their sub-dimensions differ by academic achievement level are presented in Table 7.

**TABLE 7:** One-Way ANOVA results for academic achievement level by time management and decision-making scales.

Scale and subdimensions	Academic achievement level	*N*	*M*	SD	*F*	*p*	*Post hoc* (Tukey HSD)
TMS overall	2.00–3.00	217	3.0068	0.5695	15.89	<0.001	a
	3.01–3.50	285	3.1991	0.48154			a, b
	3.51–4.00	110	3.3327	0.54657			b
	Total	612	3.1549	0.53862			
TMS time planning	2.00–3.00	217	2.9651	0.7195	14.5	<0.001	a
	3.01–3.50	285	3.1743	0.63049			a, b
	3.51–4.00	110	3.3818	0.72152			b
	Total	612	3.1375	0.69468			
TMS time attitudes	2.00–3.00	217	3.0862	0.60671	2.48	0.087	–
	3.01–3.50	285	3.207	0.61557			
	3.51–4.00	110	3.1649	0.58732			
	Total	612	3.1566	0.60899			
TMS time wasters	2.00–3.00	217	3.0346	0.80469	14.7	<0.001	a
	3.01–3.50	285	3.2842	0.59776			a, b
	3.51–4.00	110	3.4295	0.58765			b
	Total	612	3.2218	0.69184			
MDMQ self-esteem	2.00–3.00	217	1.4032	0.47043	0.75	0.474	–
	3.01–3.50	285	1.4538	0.45398			
	3.51–4.00	110	1.4303	0.45061			
	Total	612	1.4316	0.45909			
MDMQ vigilance	2.00–3.00	217	1.5899	0.41449	2.29	0.103	–
	3.01–3.50	285	1.6462	0.38338			
	3.51–4.00	110	1.6803	0.36272			
	Total	612	1.6324	0.392			
MDMQ buckpassing	2.00–3.00	217	0.7442	0.53288	1.87	0.155	–
	3.01–3.50	285	0.6871	0.52133			
	3.51–4.00	110	0.6424	0.54034			
	Total	612	0.7029	0.52963			
MDMQ procrastinatory	2.00–3.00	217	0.9124	0.5912	2.1	0.124	–
	3.01–3.50	285	0.8337	0.57506			
	3.51–4.00	110	0.7855	0.5286			
	Total	612	0.8529	0.57391			
MDMQ hypervigilance	2.00–3.00	217	0.941	0.55829	0.58	0.560	–
	3.01–3.50	285	0.8884	0.55364			
	3.51–4.00	110	0.9255	0.55907			
	Total	612	0.9137	0.55589			

TMS, Time Management Scale; MDMQ, Melbourne Decision-Making Questionnaire. Means sharing the same superscript do not differ significantly at *p* < 0.05 (Tukey HSD *post-hoc* test).

Examining Table 7, it can be seen that the total score of the Time Management Scale [*F*(2, 609) = 15.89, *p* < 0.001, η^2^ = 0.050], as well as the time planning [*F*(2, 609) = 14.50, *p* < 0.001, η^2^ = 0.045] and time wasters [*F*(2, 609) = 14.70, *p* < 0.001, η^2^ = 0.046] subdimensions, differ significantly across levels of academic achievement. In both the total score and these two subdimensions, time management scores tended to be higher among participants with higher academic achievement. However, although the between-group differences are statistically significant, the effect sizes are small, suggesting that the practical significance of these differences is limited.

None of the MDMQ subdimensions differed significantly across levels of academic achievement (*p* > .05).

### 3.7 One-Way ANOVA test results of TMS and MDMQ sub-dimension scores by social media usage

The findings of the One-Way ANOVA test conducted to examine the difference between the TMS and MDMQ sub-dimensions in relation to social media usage are presented in Table 8.

**TABLE 8:** ANOVA results for time management and decision-making styles by social media usage duration (*N* = 612).

Scale and subscales	Social media use	n	M	SD	F	P *post-hoc* (Tukey HSD)
**TMS**						
Overall	≤2 h	149	3.38	0.55	19.99	<0.001 a
	2–4 h	249	3.18	0.48		a, b
	4–6 h	155	3.02	0.52		b
	>6 h	59	2.85	0.57		c
Planning	≤2 h	149	3.36	0.71	12.35	<0.001 a
	2–4 h	249	3.18	0.64		a, b
	4–6 h	155	2.97	0.66		b
	>6 h	59	2.86	0.74		b
Attitudes	≤2 h	149	3.41	0.63	17.89	<0.001 a
	2–4 h	249	3.14	0.55		b
	4–6 h	155	3.07	0.57		b
	>6 h	59	2.80	0.65		c
Time wasters	≤ 2 h	149	3.38	0.68	8.31	<0.001 a,
	2–4 h	249	3.27	0.68		a, b
	4–6 h	155	3.11	0.64		b
	>6 h	59	2.93	0.77		c
**MDMQ**						
Self-Esteem	≤2 h	149	1.53	0.40	6.39	<0.001 a, b, c
	2–4 h	249	1.43	0.48		a, b, c
	4–6 h	155	1.42	0.39		a, b, c
	>6 h	59	1.23	0.61		d
Vigilant	≤2 h	149	1.74	0.29	8.26	<0.001 a, b
	2–4 h	249	1.65	0.38		a, b
	4–6 h	155	1.54	0.44		b
	>6 h	59	1.55	0.46		b
Buckpassing	≤2 h	149	0.59	0.48	7.89	<0.001 a
	2–4 h	249	0.69	0.54		a, b
	4–6 h	155	0.74	0.48		b
	>6 h	59	0.97	0.64		c
Procrastinative	≤2 h	149	0.72	0.55	7.59	<0.001 a
	2–4 h	249	0.85	0.56		a, b
	4–6 h	155	0.89	0.56		b
	>6 h	59	1.13	0.64		c
Hypervigilance	≤2 h	149	0.79	0.54	6.88	<0.001 a
	2–4 h	249	0.90	0.55		a, b
	4–6 h	155	0.96	0.53		B
	>6 h	59	1.15	0.62		C

TMS, Time Management Scale; MDMQ, Melbourne Decision-Making Questionnaire. *Post hoc* letters indicate significant differences (groups sharing the same letter do not differ significantly).

Examining Table 8, statistically significant differences between groups were observed for the overall mean score of the Time Management Scale [*F*(3, 608) = 19.994, *p* < 0.001, η^2^ = 0.090], as well as for the time planning subdimension [*F*(3, 608) = 12.345, *p* < 0.001, η^2^ = 0.057], the time attitudes subdimension [*F*(3, 608) = 17.889, *p* < 0.001, η^2^ = 0.081], and the time wasters subdimension [*F*(3, 608) = 8.308, *p* < 0.001, η^2^ = 0.039].

Statistically significant group differences were also found for the self-esteem subdimension of the MDMQ [*F*(3, 608) = 6.388, *p* < 0.001, η^2^ = 0.031], the vigilant subdimension [*F*(3, 608) = 8.257, *p* < 0.001, η^2^ = 0.039], the buckpassing subdimension [*F*(3, 608) = 7.894, *p* < 0.001, η^2^ = 0.037], the procrastination subdimension [*F*(3, 608) = 7.588, *p* < 0.001, η^2^ = 0.036], and the hypervigilant (panic) subdimension [*F*(3, 608) = 6.879, *p* < 0.001, η^2^ = 0.033]. Overall, scores on the buckpassing, procrastination and hypervigilant (panic) subdimensions tended to be higher among participants with longer social media usage, whereas the opposite pattern was observed for the self-esteem and vigilant subdimensions.

### 3.8 Findings on the correlations among the variables examined in the study

The correlation findings of the scales used for the variables examined in the study are presented in Table 9.

**TABLE 9:** Correlation findings of social media usage, TMS, and MDMQ.

Variables	1	2	3	4	5	6	7	8	9	10
1. Social media usage	1	–	–	–	–	–	–	–	–	–
2. TMS total	-0.299*	1	–	–	–	–	–	–	–	–
3. Time planning	-0.238*	0.933*	1	–	–	–	–	–	–	–
4. Time attitudes	-0.272*	0.681*	0.428*	1	–	–	–	–	–	–
5. Time wasters	-0.197*	0.459*	0.228*	0.321*	1	–	–	–	–	–
6. MDMQ self-esteem	-0.158*	0.414*	0.285*	0.551*	0.180*	1	–	–	–	–
7. MDMQ vigilant	-0.186*	0.387*	0.397*	0.218*	0.103*	0.210*	1	–	–	–
8. MDMQ buckpassing	0.183*	-0.368*	-0.249*	-0.470*	-0.208	-0.685*	-0.104*	1	–	–
9. MDMQ procrastinative	0.178*	-0.402*	-0.235*	-0.557*	-0.313*	-0.597*	-0.021	0.676*	1	–
10. MDMQ hypervigilance	0.176*	-0.324*	-0.178*	-0.521*	-0.183*	-0.681*	0.008	0.683*	0.715*	1

*N* = 612. *p < 0.05.

Examining Table 9, social media use shows negative and significant correlations with the overall time management score and all of its subdimensions.

Social media use was also found to be positively associated with the avoidant, procrastination and hypervigilant (panic) decision-making subdimensions, and negatively associated with the vigilant subdimension. This pattern indicates that higher levels of social media use tend to co-occur with greater tendencies toward indecision and procrastination.

The table also shows positive associations between the overall time management score and decision-making styles. Individuals with better time management tend to report higher self-esteem and a stronger tendency toward vigilant decision making. In contrast, time management is negatively associated with the avoidant, procrastination and hypervigilant (panic) subdimensions of the decision-making scale.

### 3.9 Regression findings between social media usage, the TMS and the sub-dimensions of the decision-making questionnaire

Simple linear regression analyses were conducted to determine the predictive effect of the social media usage (SMU) variable on the Time Management Scale (TMS) and the sub-dimensions of the Melbourne Decision-Making Questionnaire (MDMQ). The findings obtained are presented in Table 10.

**TABLE 10:** Results of regression analysis on the prediction of time management and decision-making styles (MDMQ) sub-dimensions by social media usage.

Independent variable	Dependent variables	*R* ^2^	*B*	*P*
SMU	Time planning	0.057	-0.18	0<0.001
	Time attitudes	0.074	-0.18	<0.001
	Time wasters	0.039	-0.15	<0.001
	MDMQ self-esteem	0.025	-0.079	<0.001
	Vigilant	0.035	-0.079	<0.001
	Buckpassing	0.034	0.106	<0.001
	Procrastinative	0.032	0.111	<0.001
	Hypervigilance	0.031	0.107	<0.001

SMU, social media usage. *p* < 0.05

Examining Table 10, social media use was found to be a significant predictor of time planning [*F*(1, 610) = 36.63, *p* < 0.001, *R*^2^ = 0.057]. Social media duration accounted for 5.7% of the variance in time planning, which corresponds to a small effect size (Cohen, 1988). Increases in social media duration were associated with lower time planning scores (β = −0.24). Social media duration also significantly predicted time attitudes [*F*(1, 610) = 48.672, *p* < 0.001, *R*^2^ = 0.074]. A one standard deviation increase in social media duration was associated with a 0.272 standard deviation decrease in time attitudes (β = −0.272), with social media explaining 7.4% of the variance in this subdimension. For the time wasters subdimension, social media duration significantly predicted scores as well [*F*(1, 610) = 24.670, *p* < 0.001]. A one standard deviation increase in social media duration was associated with a 0.197 standard deviation decrease in time wasters (β = −0.197), with social media accounting for only 3.9% of the variance. Overall, these results indicate that although the associations are statistically significant (*p* < 0.001), the corresponding effect sizes are small.

Social media use was also found to significantly predict all subdimensions of the MDMQ (*p* < 0.001). For the self-esteem subdimension, the regression model was significant [*F*(1, 610) = 15.623, *p* < 0.001, *R*^2^ = 0.025]. A one standard deviation increase in social media duration was associated with a 0.158 standard deviation decrease in self-esteem scores (β = -0.158), with social media duration accounting for only 2.5% of the variance in self-esteem. For the vigilant decision-making subdimension, the model was likewise significant [*F*(1, 610) = 21.878, *p* < 0.001, *R*^2^ = 0.035]. A one standard deviation increase in social media duration was associated with a 0.186 standard deviation decrease in vigilant decision making (β = −0.186), with social media explaining 3.5% of the variance. Regarding the buckpassing subdimension, social media use significantly predicted scores [*F*(1, 610) = 21.254, *p* < 0.001, *R*^2^ = 0.034]. A one standard deviation increase in social media duration was associated with a 0.183 standard deviation increase in buckpassing (β = 0.183), and social media explained 3.4% of the variance in this subdimension. For the procrastination subdimension, the regression model was also significant [*F*(1, 610) = 19.915, *p* < 0.001, *R*^2^ = 0.032]. A one standard deviation increase in social media duration was associated with a 0.178 standard deviation increase in procrastination (β = 0.178), with social media accounting for 3.2% of the variance. Finally, for the hypervigilant (panic) subdimension, the model was significant as well [*F*(1, 610) = 19.517, *p* < 0.001, *R*^2^ = 0.031]. A one standard deviation increase in social media duration was associated with a 0.176 standard deviation increase in hypervigilant (panic) decision making (β = 0.176), with social media explaining 3.1% of the variance in this subdimension. Overall, these findings indicate that although the effects of social media duration on decision-making subdimensions are statistically significant, the corresponding effect sizes are small.

## 4 Discussion

This study examined the relationships between social media use duration and time management and decision-making styles among individuals aged 18–36. The findings indicate that social media use differs significantly by age group: participants aged 18–23 reported substantially higher social media use than those aged 24–36, a pattern consistent with (Zia and Malik (2019).

Time management scores also tended to increase with age. Parallel findings in the literature (Başak et al., 2008; Trueman and Hartley, 1996), similarly suggest that older participants report more effective time management. With respect to decision-making, participants aged 24–36 reported higher vigilance and lower hypervigilance than those aged 18–23, which may reflect developmental differences in how individuals structure decisions under uncertainty.

Although social media use differed significantly by gender, the effect size was small and should be interpreted with caution, particularly in light of the unbalanced ratio of women to men in the sample. In contrast, significant gender differences emerged in the overall time management score and subscales. Female participants scored higher than males on time planning and dealing with time wasters, whereas males scored higher on time attitudes (evaluation of time). Prior studies have suggested that women tend to adopt a more planful approach to time management (Demirtaş and Özer, 2007), whereas men are more likely to engage in riskier behaviors (Byrnes et al., 1999). In line with our results, Erdul (2005) reported higher scores for female students than males on overall time management and time planning. Alay and Koçak (2003), similarly found gender differences favoring women in overall time management and the time planning subscale among university students, with no differences in time attitudes and time wasters.

For the decision-making measure, women obtained significantly lower decision self-esteem and higher hypervigilance scores than men. Some studies using the same instrument have reported no gender differences in self-esteem (Avşaroğlu, 2007; Can, 2009). and a meta-analysis by Zuckerman et al. (2016) identified only minimal gender differences in self-esteem. The mixed evidence regarding gender differences in self-esteem may be related to sample characteristics, measurement contexts or imbalances in female–male ratios across studies. Our finding of higher hypervigilance scores among women is consistent with Can (2009), who reported higher hypervigilance scores for female students, and with Tatlılıoğlu (2014), who likewise found significantly higher hypervigilance scores among women. Conversely, other studies have reported no gender differences in hypervigilance (Avşaroğlu, 2007; Kelecek et al., 2013), suggesting that this pattern may be context-dependent.

While social media use and decision making styles did not differ significantly by academic achievement, we observed a positive association between academic achievement and time management skills (Macan et al. (1990) proposed that time management behaviors (e.g., goal setting) are related to perceived control, which in turn is associated with beneficial outcomes such as reduced stress and better performance. Consistent with the present results, similar patterns have been reported in previous research (Britton and Tesser, 1991; Alay and Koçak, 2003; Erdem et al., 2005; Demirtaş and Özer, 2007; Başak et al., 2008). Alay and Koçak (2003) found a positive relationship between academic achievement and overall time management and a negative relationship with time wasters, but no relationship with time attitudes. Andıç (2009) reported significant relationships between academic achievement and time planning and total time management, as well as a gender difference in time attitudes favoring female students.

In the present findings, the absence of significant differences in decision-making styles according to academic achievement may indicate that decision-making is more closely related to other individual characteristics, such as personality traits, although some studies have reported associations between academic achievement and rational decision-making (Bala et al., 2017).

The comparisons of time management and decision-making across social media use durations revealed an inverse association between social media use and time management. Prior work on social media and time management is broadly consistent with this pattern, reporting that higher social media use co-occurs with weaker time management skills (e.g., Katsitadze et al., 2025). Ezeonwumelu et al. (2021) reported that Facebook and Twitter addiction was associated with poorer time management among students, and Liu et al. (2022) described a negative association between smartphone addiction and time management. In the present regression analyses, longer social media use emerged as a significant negative predictor of the time planning, time attitudes and time wasters subscales. These results suggest that higher levels of social media use tend to be accompanied by lower time management scores; however, the effect sizes were small, and the amount of variance explained was modest, indicating that these associations should be interpreted with caution.

We also found positive associations between social media use and the buckpassing, procrastinating, and hypervigilance dimensions of the MDMQ. This pattern parallels evidence of a link between social media addiction and academic procrastination (Gür et al., 2018). Another finding was that greater social media use was associated with lower decision self-esteem and lower vigilance (careful decision-making). Rosenthal and Tobin (2023) In line with this finding, Rosenthal and Tobin (2023) reported that longer time spent on social media attenuates the positive effect of self-esteem on depressive symptoms, thereby being associated with less favorable psychological well-being. This result suggests that the duration of social media use may indirectly be related to individuals’ psychological health.

The regression analyses further indicated inverse associations between social media use and decision self-esteem and vigilant decision-making, along with positive associations with buckpassing, procrastination and hypervigilance styles. These patterns are consistent with evidence of a negative relationship between addictive social media use and decision self-esteem (Andreassen et al., 2017) and with İnal’s (2023) finding that personality traits mediate the relationship between social media and decision-making. Overall, the effects of social media use on the decision-making subscales were statistically significant but small in magnitude, suggesting that social media use is only one of multiple factors associated with these decision-making tendencies.

In the context of increasing social media use, further research is needed to examine time management and decision-making styles jointly. Much of the existing work on these topics is based on findings from studies conducted in the United States and European countries. In this regard, the present study, carried out in Türkiye, contributes to the literature by providing evidence from a different cultural context and may help to broaden the generalizability of findings on these variables.

In light of the present results, it may be advisable for students to limit the time they spend on social media in order to support their academic achievement, to pay particular attention to this issue while studying, to increase their awareness of the time they devote to social media, and to use strategies such as taking planned breaks. Within school settings, psychoeducational programs designed to strengthen students’ time management and decision-making skills may also assist them in regulating their social media use.

To clarify the nature of the relationships between social media use duration and time management and decision-making styles, future studies are recommended to employ longitudinal research designs. In addition, experimental studies testing the effectiveness of interventions aimed at reducing social media use would provide valuable evidence.

### 4.1 Limitations

This study has several limitations that should be taken into account when interpreting the findings. First, the research employed a cross-sectional design, which does not allow for conclusions about causal relationships between social media use, time management and decision-making styles. Although regression analyses modeled social media use as a predictor, the associations observed cannot be interpreted as evidence of causality.

Second, social media use duration was measured via a single self-report item in the personal information form. Self-reported usage may be subject to recall bias and socially desirable responding, and may therefore not fully reflect participants’ actual usage patterns. Future studies should consider employing validated, multi-item instruments of social media use (or objective usage indicators where possible) to enhance the reliability and validity of findings.

Third, one of the subscales of the time management instrument, the “time wasters” subscale, demonstrated relatively low reliability in this study, as it did in the original Turkish adaptation. This limitation may have reduced the precision of estimates involving this dimension; accordingly, results related to this subscale should be interpreted with caution.

In addition, the effect sizes observed in the analyses and the predictive power of the regression models were generally small, indicating that social media use explained only a modest proportion of the variance in time management and decision-making variables. This suggests that the practical impact of these associations in real-world settings may be limited and that other unmeasured factors are likely to play a substantial role. Future research could examine potential mediators and moderators (e.g., personality traits, self-control, academic stress) that may amplify or attenuate the associations between social media use, time management and decision-making styles.

Furthermore, the unequal distribution of women and men in the sample may have influenced gender-related findings, and more balanced samples are needed in subsequent studies to allow for more robust examinations of gender differences. Finally, the study was conducted with university students and young adults in Türkiye; as a result, the findings may not be generalizable to other age groups or cultural contexts. Replication in more diverse and cross-cultural samples would be valuable.

The datasets used and/or analysed during the current study are available from the corresponding author on reasonable request.

Cognitive Reflection Test–Verbal Form (CRT-V): The Cognitive Reflection Test–Verbal Form (CRT-V), developed by Sirota et al. (2021) and adapted into Turkish by Şahin (2024), was also administered in the study; however, the findings related to this scale are not reported in this article.

## Data Availability

The raw data supporting the conclusions of this article will be made available by the authors, without undue reservation.

## References

[B1] Alay S. Koçak S. (2002). Validity and reliability of time management questionnaire. *Hacettepe Üniver. Eğitim Fakültesi Dergisi* 22 9–13. 10.1177/014572179902500411 10614263

[B2] Alay S. Koçak S. (2003). Üniversite öğrencilerinin zaman yönetimleri ile akademik başarıları arasındaki ilişki. *Kuram Uygulamada Eğitim Yönetimi Dergisi* 35 326–335.

[B3] Ali H. F. M. Mousa M. A. Atta M. H. R. Morsy S. R. (2024). Exploring the association between internet addiction and time management among undergraduate nursing students. *BMC Nurs.* 23:632. 10.1186/s12912-024-02273-5 39256720 PMC11389558

[B4] Andıç H. (2009). *Üniversite öğrencilerinin zaman yönetimi becerileri ile akademik başarıları arasındaki ilişki.* Yüksek lisans tezi, Afyon: Afyon Kocatepe Üniversitesi.

[B5] Andreassen C. S. Pallesen S. Griffiths M. D. (2017). The relationship between addictive use of social media, narcissism, and self-esteem: Findings from a large national survey. *Add. Behav.* 64 287–293. 10.1016/j.addbeh.2016.03.006 27072491

[B6] Aslan M. Bakır A. A. Erkuş Z. U. (2020). Öğretmen adaylarının zaman yönetimi becerileri ile akademik öz-yeterlik algıları arasındaki ilişki. *Kahramanmaraş Sütçü İmam Üniversitesi Eğitim Dergisi* 2 1–14.

[B7] Avşaroğlu S. (2007). *Üniversite öğrencilerinin karar vermede özsaygı, karar verme ve stres başa çıkma stillerinin benlik saygısı ve bazı değişkenler açısından incelenmesi.* Yayımlanmış doktora tezi, Konya: Selçuk Üniversitesi.

[B8] Bala I. Kaur R. Singh S. (2017). Decision-making styles and academic achievement. *Decision-making* 2 97–100.

[B9] Bandura A. (1991). Social cognitive theory of self-regulation. *Organ. Behav. Hum. Decis. Proc.* 50 248–287. 10.1016/0749-5978(91)90022-L

[B10] Başak T. Uzun S. Arslan F. (2008). Hemşirelik yüksek okulu öğrencilerinin zaman yönetimi becerileri. *TAF Prevent. Med. Bull.* 7 429–434.

[B11] Britton B. K. Tesser A. (1991). Effects of time-management practices on college grades. *J. Educ. Psychol.* 83 405–410. 10.1037/0022-0663.83.3.405

[B12] Byrnes J. P. (2002). The development of decision-making. *J. Adolescent Health* 31 208–215. 10.1016/S1054-139X(02)00503-7 12470917

[B13] Byrnes J. P. Miller D. C. Schafer W. D. (1999). Gender differences in risk taking: A meta-analysis. *Psychol. Bull.* 125 367–383. 10.1037/0033-2909.125.3.367

[B14] Can Ö (2009). *Üniversite öğrencilerinin akılcı olmayan inançları ve karar verme stillerinin incelenmesi.* Yüksek lisans tezi, Konya: Selçuk Üniversitesi.

[B15] Chukwu C. L. Aroh P. N. Ozor T. O. Ugwoezuonu A. U. Ezema L. C. (2022). Influence of social media usage on time management of social science education students in Nigerian Tertiary Institutions. *Webology* 19 1077–1095.

[B16] Cohen J. (1988). *Statistical power analysis fort he behavioral sciences (2nd ed.)*. Hillsdale, NJ: Erlbaum.

[B17] Demirtaş H. Özer N. (2007). Öğretmen adaylarının zaman yönetimi becerileri ile akademik başarıları arasındaki ilişki. *Eğitimde Politika Analizleri Stratejik Araştırmalar Dergisi* 2 34–47.

[B18] Deniz M. E. (2004). Üniversite öğrencilerinin karar verme stilleri ile problem çözme becerileri arasındaki ilişki. *Eğitim Araştırmaları Dergisi* 15 23–31.

[B19] Eisenberg N. Valiente C. Eggum N. D. (2010). Self-regulation and school readiness. *Early Educ. Dev.* 21 681–698. 10.1080/10409289.2010.497451 21234283 PMC3018834

[B20] Erdem R. Pirinçci E. Dikmetaş E. (2005). Üniversite öğrencilerinin zaman yönetimi Davranışları ve bu davranışların akademik başarı ile ilişkisi. *Manas Üniversitesi Sosyal Bilimler Dergisi* 7 167–177.

[B21] Erdul G. (2005). *Üniversite öğrencilerinin zaman yönetimi becerileri ile kaygı düzeyleri arasındaki İlişki.* Yüksek lisans tezi, Bursa: Uludağ Üniversitesi.

[B22] Ezeonwumelu V. U. Nwikpo M. N. Okoro C. C. (2021). Social media addiction and time management skills of university students in Akwa Ibom State, Nigeria. *Global J. Soc. Sci. Stud.* 7 24–34. 10.20448/807.7.1.24.34

[B23] Gür S. H. Bakırcı Ö Karabaş B. N. Bayoğlu F. Atli A. (2018). Üniversite öğrencilerinin sosyal medya bağımlılığının akademik erteleme davranışları üzerindeki etkisi. *İnönü Üniversitesi Eğitim Bilimleri Enstitüsü Dergisi* 5 68–77. 10.29129/inujgse.466534

[B24] Gürbüz M. Aydın A. H. (2012). Zaman kavramı ve yönetimi. *Kahramanmaraş Sütçü İmam Üniversitesi Sosyal Bilimler Dergisi* 9 1–20.

[B25] İnal H. (2023). Z kuşağında sosyal medyanın karar verme davranışları üzerine etkisi ve kişilik özelliklerinin aracılık rolü. *Soc. Sci. Stud. J.* 108 5496–5508. 10.29228/sssj.67893

[B26] Karasar N. (2017). *Bilimsel araştırma yöntemi.* Turkey: Basım, Nobel.

[B27] Karslı M. D. (2004). *Sınıfta öğrenme zamanının yönetimi, Sınıf Yönetimi.* Turkey: Pegem A Yayıncılık.

[B28] Katsitadze N. Kharadze N. Pirtskhalaishvili D. (2025). The impact of social media on students’ effective time management. *Baltic J. Econ. Stud.* 11 200–210. 10.30525/2256-0742/2025-11-1-200-210

[B29] Kelecek S. Altıntaş A. Aşçı F. H. (2013). Sporcuların karar verme stillerinin belirlenmesi. *CBÜ Beden Eğitimi Spor Bilimleri Dergisi* 8 21–27.

[B30] Kuşcu Karatepe H. Özcan Yüce U. Öztürk Yıldırım T. (2020). Zaman yönetimi: Üniversite öğrencileri üzerinde bir araştırma. *Bartın Üniversitesi İktisadi İdari Bilimler Fakültesi Dergisi* 11 1–21.

[B31] Liu F. Xu Y. Yang T. Li Z. Dong Y. Chen L. (2022). The mediating roles of time management and learning strategic approach in the relationship between smartphone addictio academic procrastination. Erratum in: *Psychol. Res. Behav. Manag.* 15, 2897–2898. 10.2147/PRBM.S391854 36148284 PMC9488603

[B32] Macan T. H. Shahani L. Dipboye R. L. Phillips A. P. (1990). College students’ time management: Correlations with academic performance and stress. *J. Educ. Psychol.* 82 760–768. 10.1037/0022-0663.82.4.760

[B33] Mann L. Burnett P. Radford M. Ford S. (1997). The melbourne decision making questionnaire: An instrument for measuring patterns for coping with decisional conflict. *J. Behav. Decision Making* 10 1–19. 10.1002/(SICI)1099-0771(199703)10:1<1::AID-BDM242<3.0.CO;2-X

[B34] Peng M. Xu Z. Huang H. (2021). How does information overload affect consumers’ online decision process? An event-related potentials study. *Front. Neurosci.* 15:695852. 10.3389/fnins.2021.695852 34744601 PMC8567038

[B35] Rosenthal S. R. Tobin A. P. (2023). Self-esteem only goes so far: The moderating effect of social media screen time on self-esteem and depressive symptoms. *Behav. Inform. Technol.* 42 2688–2695. 10.1080/0144929X.2022.2139759 37994349 PMC10662696

[B36] Şahin G. (2024). Bilişsel yansima testi sözel formunun türkçe’ye uyarlanması. *Edebiyat Beşeri Bilimler Dergisi* 57–67. 10.55590/literatureandhumanities.1462833

[B37] Sarıkaya Aydın K. Koçak S. (2016). Üniversite öğrencilerinin zaman yönetimi becerileri ile akademik erteleme düzeylerinin incelenmesi. *Uşak Üniversitesi Eğitim Araştırmaları Dergisi* 2 17–38. 10.29065/usakead.256378

[B38] Sirota M. Dewberry C. Juanchich M. Valus L. Marshall A. C. (2021). Measuring cognitive reflection without maths: Development and validation of the verbal cognitive reflection test. *. Behav. Decis. Mak.* 34, 322–343. 10.1002/bdm.2213

[B39] Tabachnick B. G. Fidell L. S. (2007). *Using multivariate statistics*, 5th Edn. Boston, MA: Pearson Education, Inc.

[B40] Tatlılıoğlu K. (2014). Üniversite öğrencilerinin karar vermede öz-saygı düzeyleri ile karar verme stilleri arasındaki ilişkinin bazı değişkenlere göre incelenmesi. *Akademik Sosyal Araştırmalar Dergisi* 2 150–170. 10.16992/ASOS.46

[B41] Trueman M. Hartley J. (1996). A comparison between the time-management skills and academic performance of mature and traditional-entry university students. *High. Educ.* 32 199–215. 10.1007/BF00138396

[B42] Varlamova V. (2008). *The relationship between time management and decision making processes.* Master’s thesis, Christchurch: University of Canterbury.

[B43] We Are Social and Meltwater (2025). *Digital 2025: The essential guide to the global state of digital.* San Francisco, CA: We Are Social and Meltwater.

[B44] Yardım T. Engin G. (2022). Sınıf eğitimi öğretmen adaylarının akademik erteleme davranışları ve zaman yönetim becerileri arasındaki ilişkinin incelenmesi. *Trakya Üniversitesi Sosyal Bilimler Dergisi* 24 347–368. 10.26468/trakyasobed.1099730

[B45] Zia A. Malik A. A. (2019). Usage of social media, age, introversion and narcissism: A correlational study. *Bahria J. Prof. Psychol.* 18:33.

[B46] Zimmerman B. J. (2002). Becoming a self-regulated learner: An overview. *Theory Into Pract.* 41 64–70. 10.1207/s15430421tip4102_2

[B47] Zuckerman M. Li C. Hall J. A. (2016). When men and women differ in self-esteem and when they don’t: A meta-analysis. *J. Res. Personal.* 64 79–98. 10.1016/j.jrp.2016.07.007

